# Editorial: Importance of root symbiomes for plant nutrition: new insights, perspectives and future challenges, volume II

**DOI:** 10.3389/fpls.2023.1296604

**Published:** 2023-10-04

**Authors:** Debatosh Das, Arjun Kafle, Tania Ho-Plágaro, Sabine D. Zimmermann, Heike Bücking, Kevin Garcia

**Affiliations:** ^1^ Division of Plant Science and Technology, University of Missouri, Columbia, MO, United States; ^2^ National Products Utilization Research Unit (NPURU), USDA-ARS, Oxford, MS, United States; ^3^ Department of Crop and Soil Sciences, North Carolina State University, Raleigh, NC, United States; ^4^ IPSiM, Univ Montpellier, CNRS, INRAE, Institut Agro, Montpellier, France

**Keywords:** arbuscular mycorrhizal symbiosis, ectomycorrhizal symbiosis, plant growth-promoting bacteria (PGPB), plant nutrition, nitrogen-fixing rhizobia

Symbiotic interactions between plant roots and soil microbes improve plant nutrient acquisition, provide a defense against pathogens, and allow plants to adapt to varying environmental conditions. Understanding the establishment of these interactions and the maintenance of root and rhizosphere microbiota by host plants and their role in plant nutrition is essential to protect ecosystems and harness these symbioses in agricultural settings (van der Heijden et al., 2008). This compilation of research articles provides a comprehensive overview of recent advancements in plant microbiota, including specific symbiotic partners like plant growth-promoting bacteria (PGPB), arbuscular mycorrhizal (AM) and ectomycorrhizal (ECM) fungi, as well as nitrogen-fixing rhizobia. Furthermore, this Research Topic explores external factors such as nutrient management strategies that affect root symbiosis efficiency, and crop intercropping that impact nutrient availability in the field.

Significant advantages of beneficial plant-microbe interactions in symbiosis are the increase of nutrient uptake and soil exploration for colonized roots. AM fungi also emerge as significant contributors to sustainable agriculture. Goddard et al. unveiled the transcriptomic and metabolomic alterations in grapevine roots and leaves after AM colonization. They reported increased levels of various compounds in AM plants, including unsaturated fatty acids in roots and leaves, salicylic and jasmonic acids, as well as pathogenesis-related proteins in roots. These changes can potentially bolster resistance to diverse pathogens in AM plants through direct or priming effects (Pieterse et al., 2014). Therefore, this study highlights the importance of symbiotic interactions between grapevine and AM fungi, particularly in the context of environmental stresses. Taking a synergistic stance, the study by Nacoon et al. adopted a multifaceted approach to enhance plant growth. They showed that colonizing sunchoke plants with AM fungi improved the overall growth, but there was limited evidence for synergistic plant growth responses when the plants were co-inoculated with the phosphorus-solubilizing bacteria (PSB) *Burkholderia vietnamiensis* strain KKUT8-1. Co-inoculation with both microbes marginally improved growth, implying that the often-cited synergy between microbial consortia was less prevalent in this particular context. The importance of AM colonization was particularly pronounced under low water conditions, supporting its role in the drought tolerance of plants. Although the significance of synergy between plants and symbiotic microbes can be observed in agroecosystems, it is still difficult to dissect and isolate the diverse contributions coming from a plethora of factors that can influence plant fitness and crop productivity, many of them acting synergistically or in opposition with each other. This is the reason why conflicting results can be obtained using different set-up, plant/cultivar, microbial partners, or environmental conditions. Consequently, this study highlighted intricate interactions between bio-inoculants and their potential to mitigate challenges in modern agriculture.

In the context of plant P nutrition, Amenc et al. investigated the impact of ECM fungi on P acquisition by *Pinus pinaster*. The study introduced a new perspective on the fungal transporter HcPT1.1 by investigating the molecular mechanisms underlying P transfer. Although still puzzling, it appeared that HcPT1.1 might be involved in P efflux towards colonized roots similar to the previously characterized phosphate transporter, HcPT2 (Becquer et al., 2018), suggesting potential functional redundancy or more complex regulatory interactions between HcPT1.1 and HcPT2 at the protein level. This report highlights the complexity of nutrient exchange processes in the ECM symbiosis and emphasizes the potential for groundbreaking insights in future research.

Transitioning to nutrient dynamics, Li et al. explored the equilibrium between nitrogen (N) and P availability in multiple legume species upon symbiotic nitrogen fixation (SNF). This study revealed that external N addition can reduce SNF by intensifying P limitations, leading to increased rhizosphere P mobilization at the expense of root biomass and carbohydrate concentrations vital for root nodule development. Additionally, the addition of P countered its limitations induced by N, reducing the suppression of SNF. Legume species with stable N:P ratios displayed lower reductions. In summary, this research identified a trade-off between rhizosphere P mobilization and SNF under varying N availability. A species-specific stoichiometric regulation in the SNF response to N addition was also demonstrated. With insights derived from various legume species, the research underscores the need for nuanced nutrient management practices to optimize legume performance in diverse contexts. Diversifying the discourse, Tang et al. delve into the potential of intercropping systems as a sustainable agricultural strategy. Analyzing the complex interplay of metabolites and soil properties within the sugarcane/peanut intercropping system, the research underscored the positive ripple effect of intercropping on soil nutrient levels and enzymatic activities. Additionally, the regulation of purine metabolism emerged as a significant factor. This regulation was instrumental in encouraging the release of adenosine and adenine from roots, leading to their accumulation within the rhizospheric soil. Intercropping will probably modify the beneficial microbiome thus contributing to nutritional amelioration (Lian et al., 2019; Saleem et al., 2019). This article bridges the gap between plant metabolic shifts and improvements in soil quality, offering novel insights into enhancing agricultural productivity while maintaining environmental balance.

Amidst the evolving landscape of climate change and its implications for agriculture, the pivotal role of beneficial microbes takes center stage (Usman et al., 2021). A mini-review by Mukherjee et al. highlighted the imperative for a deeper comprehension of the genetic mechanisms driving PGPB-induced plant growth responses in host plants. Transcriptomic analyses provided a glimpse into the intricate orchestration of plant responses during PGPB interactions, shedding light on gene expression patterns linked to defense, flavonoid synthesis, receptor-like kinases, hormone regulation, and nutrient assimilation. This paves the way for targeted genetic and biochemical discoveries, potentially unlocking the full spectrum of plant-PGPB interactions.

The studies presented in this Research Topic display a compelling narrative of the interconnectedness between plants and their associated soil microbes. They illuminate these relationships’ genetic, metabolic, and ecological facets, unveiling their potential for sustainable agriculture amidst the challenges posed by climate change. Through deciphering the intricate mechanisms governing these interactions, this body of research contributes significantly to optimizing plant-microbe partnerships, thereby forging a path toward resilient and productive agricultural systems.

## Author contributions

DD: Writing – original draft. AK: Writing – review & editing. TH-P: Writing – review & editing. SZ: Writing – review & editing. HB: Writing – review & editing. KG: Writing – review & editing.
